# The effect of cranial techniques on the heart rate variability response to psychological stress test in firefighter cadets

**DOI:** 10.1038/s41598-023-34093-z

**Published:** 2023-05-13

**Authors:** Małgorzata Wójcik, Idzi Siatkowski

**Affiliations:** 1 Department of Physiotherapy, Poznan University of Physical Education, Faculty of Sport Sciences in Gorzow Wlkp., 61-871 Poznan, Poland; 2 Department of Mathematical and Statistical Methods, Poznan University of Life Science, 60-637 Poznan, Poland

**Keywords:** Cardiology, Quality of life

## Abstract

Heart rate variability (HRV) is a simple tool to monitor cardiovascular stress. The proper function of the cardiovascular system is a problem among firefighters. Physical activity has health benefits correlated with psychological stress. Physically active people should be more resilient to psychological stress, but this has not always been demonstrated. The aim of this study was to determine whether cranial techniques would have an effect on HRV parameters. Osteopathy in the cranium reduces stress and improves cardiovascular function. Fifty-seven firefighter cadets aged 18–24 years (21.63 ± 1.41) participated in the study. All subjects had their heart rate variability measured and were randomly assigned either to the cranial techniques (CS) group, with therapy performed once a week for 5 weeks), or to the control group (CO). After 5 weeks, heart rate variability was measured again in both groups. In the Friedman test, in the CS group there was a statistically significant effect of cranial techniques on Heart Rate (HR) and Low Frequency (LF), but not on High Frequency (HF); in the CO group, a statistically significant difference was observed for HR, HF and LF. In the Nemenyi test, in the CS group there was a statistically significant difference for HR and LF and in the CO group for HR, HF and LF. After applying hierarchical clustering with Euclidean measure and the complete method, dendrograms were drawn up showing similarities for HR, HF and LF values. The cranial techniques and touch might exert a beneficial effect on HRV. Both factors can be used in stressful situations to lower HRV.

## Introduction

The autonomic nervous system (ANS), which influences the heart rate HR) in short-time intervals, is subdivided into two distinct components, namely the sympathetic nervous system (SNS) and parasympathetic nervous system (PNS). The SNS, known as the quick response system, predominates during elevated activity and stressful states and its stimulation results in an increase in HR. The PNS, known as the relaxed response system, predominates in the quiet and relaxing states, and is related to a decrease in HR. In healthy individuals, the two systems work together (promptly alternating between each other) to maintain the regulatory balance in physiological autonomic function. Autonomic function can be measured non-invasively from physiologic signals of heart rate, respiratory rate, and blood pressure. Heart Rate Variability (HRV), or the fluctuation in the length of time between heart beats (R-R intervals), provides a measure of sympathetic and parasympathetic interplay, and therefore ANS functional maturation^1^. High-frequency (HF) variability reflects parasympathetic function and is influenced by the respiratory rate, while low-frequency (LF) variability is due to a combination of sympathetic and parasympathetic inputs and baroreflex-induced changes in heart rate^2^.

The professional activities performed by firefighters require continuous regulation of the cardiovascular system, with the predominant modulation of the sympathetic nervous system (SNS)^3^. The proper functioning of the cardiovascular system among firefighters is problematic^4^. Excessive stress on the cardiovascular system among this group has led to sudden cardiac deaths (SCDs)^5^. Participation in firefighting operations causes high levels of stress that negatively affect the cardiovascular system^6^, thus causing a risk of cardiovascular disease in this occupational group^7^. The occupation of a firefighter is fraught with cardiac autonomic activity and sleep disorders^8^.

Osteopathic craniosacral therapy involves the use of gentle and specialised techniques to minimise tension in the skull, pelvis, diaphragm, chest and sacrum. The therapy leads to the relaxation of connective tissue structures whose tension is usually the cause of health problems. In addition, it improves the functioning of the cardiovascular system and eliminates somatic complaints such as migraines or muscle pain^9–11^.

To prevent cardiovascular diseases among occupations high in stress, it would be advisable to find non-invasive treatment methods/techniques aimed at reducing stress levels, i.e. effects on HRV. A simple tool to monitor cardiovascular stress is the HRV assessment^12–15^, which is considered a non-invasive method to assess the functioning of the autonomic nervous system (ANS)^5^. Osteopathic manipulative therapy (OMT) has a positive effect on cardiovascular autonomic parameters^16,17^.

Prior to the study, the following research hypothesis was formulated: craniosacral therapy significantly affects HRV parameters (causes them to decrease and/or increase) in male firefighter cadets.

In addition, the aim of the study was to check whether craniosacral therapy could be a low-cost, non-invasive and non-pharmacological way to reduce HRV values. It was also decided to check whether touch (holding the head in the placebo group) could have an effect on HRV values.

## Materials and methods

### Design

The research was conducted as part of the project “Craniosacral therapy in stress reduction”. Experimental sessions were conducted between 9:30 a.m. and 1:00 p.m., lasting about 50 min and consisting of 3 parts: Phase 1—participants were exposed to a mental stressor and an osteopathic or placebo session of 20 min. Phase 2 was homologous to Phase 1. Both Phases incorporated baseline (first 5 min), arithmetic task (5 min) and post-stress (5 min) intervals. To determine the effects of stress on the participant's heart rate (HR), respiration rate (RR) and skin conductance (SC), values were continuously recorded during phase 1 and phase 2. After 5 min at baseline, an arithmetic test was administered to induce mental workload and psychological stress. For the math stressor, participants performed a series of subtraction problems aloud, continuously for 5 min, and were asked to give answers aloud as fast as possible. They were instructed that the experimenter would correct any errors they made and that they should then continue from the correct number to maintain maximal stress from task involvement and moderate stress task difficulty (approximately 10 correct answers per minute^18,19^. Better performance led to more difficult math problems in the subsequent minute. Following the arithmetic task, participants were asked to sit still for a 5-min recovery period.

The first measurement was taken before starting craniosacral therapy and administering the placebo (9:30 a.m. and 1:00 p.m.). The second measurement was taken after 5 weeks of craniosacral therapy and placebo, at the same hour as the first measurement.

### Sample

The study participants consisted of Fire Service cadets from the State Fire Service College. Fifty-seven cadets (30-man group with craniosacral therapy, 27-man group with placebo) aged 18–24 (21.63 ± 1.41), with a mean Body Mass Index of 24.44 ± 3.05 kg/m^2^, voluntarily participated in the study. Participants were recruited through meetings organized at the firefighting academy, as well as posters and leaflets. All participants were briefed on the study protocol using an information sheet, and their informed written consent for participating in the study was obtained. Interested candidates were screened by interview to check their eligibility according to the inclusion and exclusion criteria.

Inclusion criteria were as follows: male, firefighter academy cadet and voluntary participation.

Exclusion criteria included any history of osteopathic therapy, daily smoking, alcohol abuse, heavy caffeine use (> 300 mg/day), medication intake, drug abuse, reported medical illness, history of endocrine disorder, psychiatric disorder and cardiovascular disease.

A random number generator was used to assign the subjects to the groups. Each participant received a number drawn for allocation to one of the study groups. The therapeutic intervention sessions were conducted once a week for five consecutive weeks, with experimental sessions conducted between 9:30 a.m. and 1:00 p.m.

### Procedures

#### Randomization

To allocate participants in groups, a block randomization was performed using an Excel file. First, a block to assign sample numbers equally to each group was generated. The block size was randomly generated (2-, 4- or 6-letter combination of A and B). Then each block was assigned to a group. Each participant was provided with the printed number, so the therapist could not predict which treatment placebo or craniosacral therapy) would be performed until the participant came.

#### Cranial techniques

Before applying osteopathic craniosacral therapy, all participants from the study group (N = 30) were acquainted with its methodology. Therapy sessions were held in a warm, quiet room, and due to the time of day (9.30 a.m. and 1.00 p.m.), no artificial light (room lighting) was used. The therapeutic intervention sessions were conducted once a week for five consecutive weeks, with experimental sessions conducted between 9:30 a.m. and 1:00 p.m. With the participants supine, the same female therapist administered the therapy each time according to the previously described procedure^20,21^. In this study, we used structural approach craniosacral therapy. Following the release of mobilised structures, the therapist applied individual phases of osteopathic craniosacral therapy (sacrum compression and traction, AO—Atlanto-occipital joint, mobilization of the frontal bone, parietal bones, sphenoid bone and temporal bones, and the final step was the CV4 technique. For a full description of the procedure, see Liem^20,21^. For the non-intervention group (N = 27), the therapist only held the subject’s head (while the subject was in the supine position) and did not use her hands for the application of any osteopathic techniques. The treatment time for both groups' individual subjects was 20 min, which was ensured by using a stopwatch. None of the 57 participants had received any osteopathic therapy before the study, and the study subjects did not have any previous knowledge or experience of the osteopathic craniosacral procedures. At the start of the study, both groups had 30 participants. Due to the illness of 3 men, the group decreased to 27 without intervention.

#### Outcomes

The primary outcomes of the current study were changes in indicators of autonomic stimulation frequency of heart contractions in response to the math task and during recovery following craniosacral/placebo therapy. Stress response is connected with acceleration and shortness of breath, which leads to an increased heart rate^22,23^. For measuring multisensory data, a ProComp Infinity 5 (Thought Technology Ltd) device was used. The device is designed to measure up to 8 biosensors. For the current experiment, 3 channels were used, including blood volume pulse (BVP)—a photoplethysmographic sensor measured on finger, with a sampling rate at 256 per s), skin conductance (SC)—two sensors measured on finger, with a sampling rate at 256 per s, (3) respiration rate (RR)—measured at abdomen with a sampling rate at 256 per s. The device was calibrated by the manufacturer and met the current guidelines of the psychophysiological measurements^22,23^. On the participant's non-dominant hand, a BVP optical pulse sensor was placed on the palmar surface of the little finger to measure heart rate (HR), and two Ag/AgCl electrodes were placed on the palmar surface of the middle and index fingers to acquire an SC signal. The respiratory transducer records respiratory frequency using a belt which measures the force of chest expansion during each inhalation and ex halation. SC, together with HR and RR can provide useful information about the activity of the sympathetic nervous system: greater values represent greater sympathetic activity^22,23^. In addition to the measurement of autonomic stress response and recovery following math stress, secondary outcomes included the baseline HR levels following the craniosacral/placebo intervention.

#### Treatment blinding

None of the participants had received any osteopathic treatment prior to the study nor did they have any knowledge about osteopathic procedures. To ensure blinding, participants did not contact one another until all measurements were collected. The randomization code was kept by the therapist and only revealed once the final results had been collected. Participants remained blinded to treatment allocations until all participants had completed the study. At the end of the intervention, participants were asked to guess which treatment they had undergone. Measurements were recorded by a psychologist who was blinded to intervention.

The following designations for heart rate variability measurements were adopted: HR—Heart Rate, LF—Low Frequency and HF—High Frequency. LF and HF were measured in ms^2^, and HR in bpm (beats per min) (Table 1).

**Table 1:** Before starting the study.

1	HR1_1 HR	Measurement before the start of the heart rate variability assessment
	LF1_1 LF	Power measurement before the start of the heart rate variability assessment
	HF1_1 HF	Power measurement before the start of the heart rate variability assessment
2	HR2_1 HR	Measurement during the heart rate variability assessment—at the beginning
	LF2_1 LF	Power measurement during the heart rate variability assessment after experiencing minor stress
	HF2_1 HF	Power measurement during the heart rate variability assessment after experiencing minor stress
3	HR3_1 HR	Measurement at the end of the heart rate variability assessment
	LF3_1 LF	Power measurement at the end of the heart rate variability assessment after experiencing great stress
	HF3_1 HF	Power measurement at the end of the heart rate variability assessment after experiencing great stress

After 5 weeks, HRV was measured in the CO group and in the CS group (after 5 weeks of cranial techniques and placebo). The following designations were used for heart rate variability (HRV) (Table 2).

**Table 2:** After completion of the study.

1	HR1_2 HR	Before the start of the heart rate variability assessment
	LF1_2 LF	Power before the start of the heart rate variability assessment
	HF1_2 HF	Power before the start of the heart rate variability assessment
2	HR2_2 HR	Measurement during the heart rate variability assessment—at the beginning
	LF2_2 LF	Power measurement during the heart rate variability assessment after experiencing minor stress
	HF2_2 HF	Power measurement during the heart rate variability assessment after experiencing minor stress
3	HR3_2 HR	Measurement at the end of the heart rate variability assessment
	LF3_2 LF	Power measurement at the end of the heart rate variability assessment after experiencing great stress
	HF3_2 HF	Power measurement at the end of the heart rate variability assessment after experiencing great stress

To determine the effect of cranial techniques on heart rate variability, the CS group (cranial techniques group) and the control group (CO) were analysed separately and independently. As the data obtained do not follow a normal distribution and are coupled data, the non-parametric Friedman test^24^ and Nemenyi test of multiple comparisons^25^ were applied. In addition, hierarchical clustering with the Euclidean measure and the complete method^26^ was used in the multivariate statistical analysis of the data^27^. The results of hierarchical clustering were presented graphically in the form of dendrograms.

### Statistics

In the first stage of the data analysis, basic descriptive statistics were compiled. Then normality of distribution was tested using the Shapiro–Wilk test. Due to the lack of normality, non-parametric Friedman test were used. Since the Friedman test showed there is a significant difference between the groups, we use the Nemenyi test to determine which groups are significantly different. Furthermore, the similarity of groups was studied by hierarchical cluster analysis based on the Euclidean measure with the complete method and the results were presented on a dendrogram. All calculations, statistical analyses and figures were performed using the R software version 4.2.2 (R Core Team, 2022)^28^.

### Ethical approval and consent to participate

All procedures performed in studies involving human participants were following the ethical standards of the institutional and/or national research committee and with the 1964 Helsinki declaration and its later amendments or comparable ethical standards. The study protocol was approved by the Bioethics Committee of the Nicolaus Copernicus University in Toruń functioning at the Collegium Medicum in Bydgoszcz (permit No. KB/99/2016).

## Results

### Basic score statistics for groups CS and CO

The basic score statistics, that is, Minimum, Maximum, Median, Mean, 1stQu and 3rdQu for Heart Rate, Low Frequency and High Frequency are shown in Table 3.

**Table 3:** Descriptive statistics for groups CS and CO (Min – minimum, 1stQu—first quartile, Median, Mean, 3rdQu—third quartile, Max—maximum).

	Min	1stQu	Median	Mean	3rdQu	Max
CS_HR1_1	55.94	64.49	70.42	74.85	80.87	113.42
CO_HR1_1	61.43	70.84	75.50	77.88	84.56	116.42
CS_HR2_1	54.93	76.64	83.34	87.18	95.06	137.24
CO_HR2_1	60.59	75.44	86.22	88.39	98.06	140.24
CS_HR3_1	55.94	66.67	73.67	76.41	81.19	120.48
CO_HR3_1	64.94	70.74	75.24	78.52	83.58	112.07
CS_HF1_1	261.60	549.90	810.10	1819.60	2286.90	10,474.00
CO_HF1_1	262.60	771.50	990.70	1214.90	1856.20	2439.00
CS_HF2_1	140.20	1323.30	1873.60	3247.70	5667.20	10,474.00
CO_HF2_1	151.20	1178.30	2984.70	3083.30	4688.80	6928.80
CS_HF3_1	191.60	749.50	1352.50	2693.20	3066.30	9006.80
CO_HF3_1	262.60	1089.00	2057.60	2546.60	3503.90	6944.30
CS_LF1_1	497.20	725.20	889.50	1263.20	1518.00	4281.80
CO_LF1_1	659.30	805.80	1011.30	1230.90	1454.90	2944.10
CS_LF2_1	301.00	1247.00	2845.00	3266.00	4895.00	11,569.00
CO_LF2_1	312.00	1785.00	2871.00	2755.00	3448.00	5161.00
CS_LF3_1	433.60	977.80	1387.70	1926.30	2077.90	5001.40
CO_LF3_1	354.10	1117.60	1894.00	1955.50	2559.00	4281.80
CS_HR1_2	58.43	67.44	70.30	72.93	79.61	98.90
CO_HR1_2	62.35	72.31	76.16	76.98	80.55	93.36
CS_HR2_2	66.24	76.14	85.19	85.73	95.35	119.51
CO_HR2_2	70.02	78.21	84.41	87.35	92.96	111.73
CS_HR3_2	64.94	71.28	75.79	77.35	84.40	98.57
CO_HR3_2	68.13	72.27	75.99	77.57	81.08	91.22
CS_HF1_2	499.10	795.70	1625.10	1521.40	1893.50	4657.80
CO_HF1_2	472.90	1152.00	1379.90	1386.90	1655.20	2556.20
CS_HF2_2	721.80	1613.00	3220.50	3235.70	4602.10	5891.10
CO_HF2_2	412,70	1448.70	3505.80	3162.10	4543.10	5863.20
CS_HF3_2	503.20	1342.90	2063.80	2633.50	3504.80	6944.30
CO_HF3_2	623.60	1406.10	2021.50	2860.70	4291.70	7463.70
CS_LF1_2	784.20	896.30	1078.60	1256.80	1545.60	2603.70
CO_LF1_2	811.50	963.80	1133.20	1260.20	1431.90	2120.30
CS_LF2_2	896.50	2404.60	2893.10	3185.30	3844.30	7099.90
CO_LF2_2	910.80	2455.50	2847.40	2947.30	3393.50	6075.50
CS_LF3_2	970.20	1211.10	1752.70	1920.20	2508.00	4021.50
CO_LF3_2	746.80	1512.30	1873.60	2062.40	2737.30	3344.60

### Analysis of the CS Group

For the CS group, in the first stage of analysis the non-parametric Friedman test for HR (Heart Rate) was applied to the coupled data and a p-value < 0.0001 was obtained. This means that there is a statistically significant difference between the compared HR values (objects). Then, for further detailed conclusions, the Nemenyi multiple test was applied and the obtained p-values are shown in Table 4. There was a statistically significant difference between: HR1_1 and HR2_2; HR2_1 HR3_1; HR2_1 and HR1_2; HR3_1 and HR2_2; HR1_2 and HR2_2.

**Table 4:** P-values of Nemenyi test of HR for CS.

	HR1_1	HR2_1	HR3_1	HR1_2	HR2_2
HR2_1	**< 0.001**	–	–	–	–
HR3_1	NS	**< 0.001**	–	–	–
HR1_2	NS	**< 0.001**	NS	–	–
HR2_2	**< 0.001**	NS	**< 0.001**	**< 0.001**	–
HR3_2	NS	NS	NS	NS	**0.029**

Values in bold—statistically significant differences, NS—no statistically significant differences.

**Figure 1:**
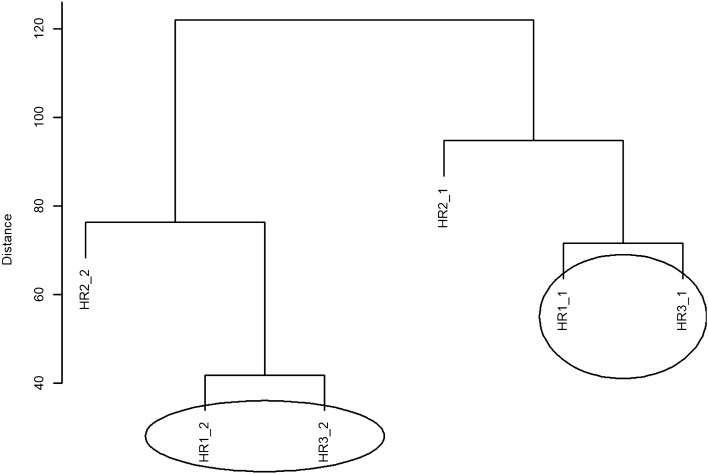
A dendrogram of similarities between the HR values in the CS group, based on hierarchical cluster analysis according to the complete clustering method.

Subsequently, hierarchical clustering with the Euclidean measure and the complete method was applied as part of the multivariate statistical analysis of the data. The results of the hierarchical clustering are presented graphically in the form of a dendrogram (Fig. 1), in which there are two groups of similar objects, i.e. groups within which similar heart rate values occur. Heart Rate HR1_2 and HR3_2 is the group in which heart rate values have the weakest effect, while the second group of similar objects consists of HR1_1 and HR3_1. HR2_2 and HR2_1 do not belong to and do not form a group of similar objects, although the effect is strongest.

**Figure 2:**
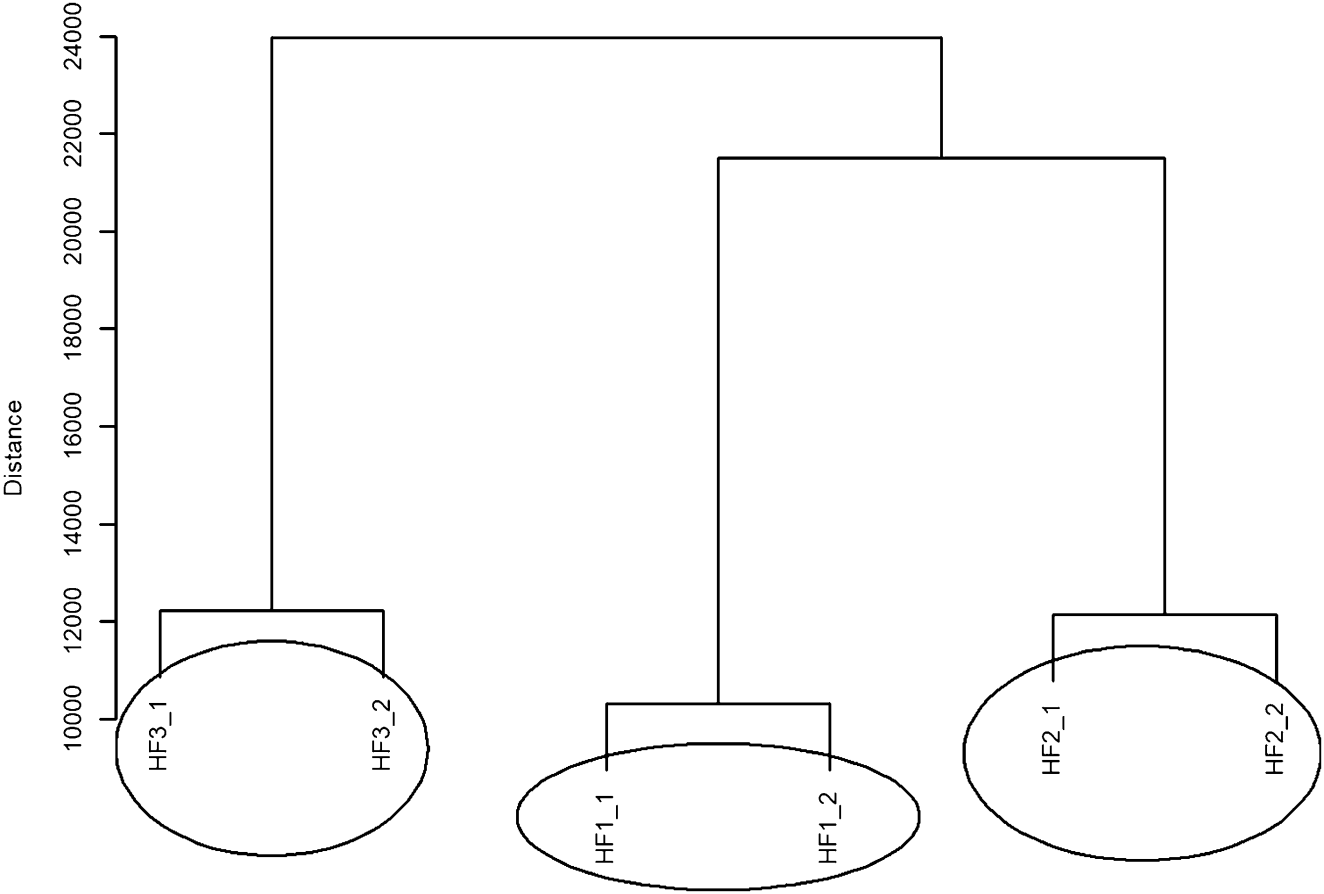
A dendrogram of similarities between the HF values in the CS group based on hierarchical cluster analysis according to the complete clustering method.

In the CS group, when analysing the data for HF (High Frequency), the non-parametric Friedman test was also applied and a p-value = 0.058 was obtained. This means that there is no statistically significant difference between the compared values of the High Frequency component of the heart rate. The dendrogram of hierarchical grouping with the Euclidean measure and the complete method is presented in Fig. 2. Three different groups containing similar objects can be distinguished, i.e. three groups containing similar High Frequency values. One group of High Frequency values rep-resents the weakest effect and contains HF1_1 and HF1_2. Another group contains HF2_1 and HF2_2 values (objects), and the last group consists of HF3_1 and HF3_2 values.

In the CS group, when analysing LF (Low Frequency) values, as with HR and HF, the non-parametric Friedman test was first applied to the coupled data and a p-value < 0.0001 was obtained. This means that there is a statistically significant difference between the compared LF values (objects). Then, for further detailed conclusions, the Nemenyi multiple test was applied and the obtained p-values are shown in Table 5. Statistically significant differences occur between: LF1_1 and LF2_2; LF1_2 and LF2_2.

**Table 5:** P-values of the Nemenyi test of LF for the CS group.

	LF1_1	LF2_1	LF3_1	LF1_2	LF2_2
LF2_1	**0.002**	–	–	–	–
LF3_1	NS	NS	–	–	–
LF1_2	NS	**0.015**	NS	–	–
LF2_2	**< 0.001**	NS	**0.007**	**< 0.001**	–
LF3_2	NS	NS	NS	NS	**0.036**

Values in bold—statistically significant differences, NS—no statistically significant differences.

**Figure 3:**
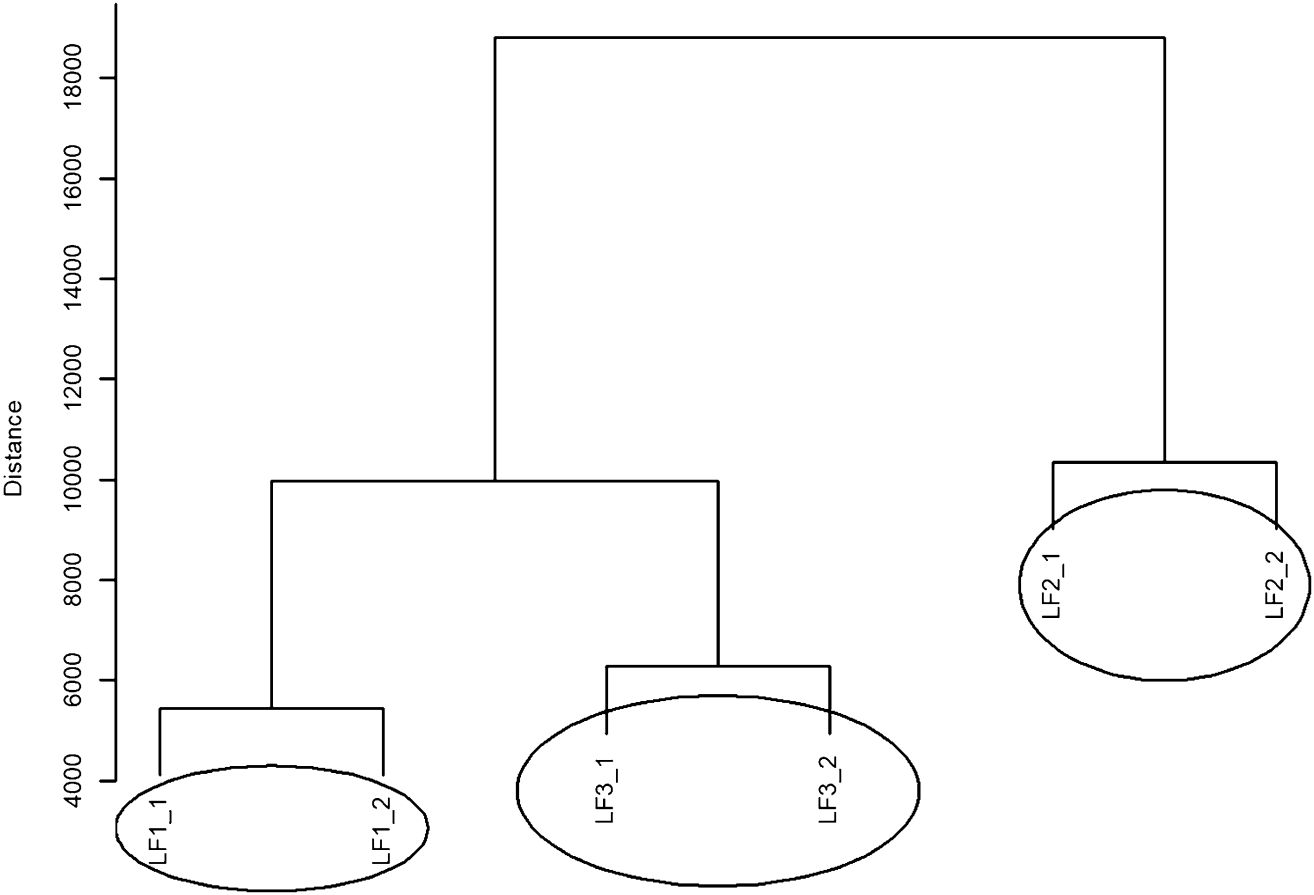
A dendrogram of similarities between the LF values in the CS group based on hierarchical cluster analysis according to the complete clustering method.

The dendrogram of hierarchical clustering with Euclidean measure and complete method for LF values in the CS group is shown in Fig. 3. Three different groups can be distinguished which contain LF values. One group of similar LF values which shows the weakest effect contains LF1_1 and LF1_2 values. The next group contains LF3_1 and LF3_2 values, and another group consists of LF2_1 and LF2_2 values.

### Analysis of the CO group

The analysis of the control group (CO) was performed analogically to the analysis of the CS group. In the first step of the analysis, the non-parametric Friedman test for HR (Heart Rate) was applied to the coupled data and a p-value < 0.0001 was obtained. This means that there is a statistically significant difference between the compared HR values. Then, the Nemenyi multiple test was applied for further detailed conclusions and the p-values obtained are presented in Table 6. Statistically significant differences occur between: HR1_1 and HR2_2; HR2_1 and HR3_1; HR2_1 and HR1_2; HR3_1 and HR2_2; HR1_2 and HR2_2; HR2_2 and HR3_2.

**Table 6:** P-values of the Nemenyi test of HR for the CO group.

	HR1_1	HR2_1	HR3_1	HR1_2	HR2_2
HR2_1	**0.004**	–	–	–	–
HR3_1	NS	**< 0.001**	–	–	–
HR1_2	NS	**< 0.001**	1	–	–
HR2_2	**< 0.001**	NS	**< 0.001**	**< 0.001**	–
HR3_2	NS	**0.005**	NS	NS	**< 0.001**

Values in bold—statistically significant differences, NS—no statistically significant differences.

**Figure 4:**
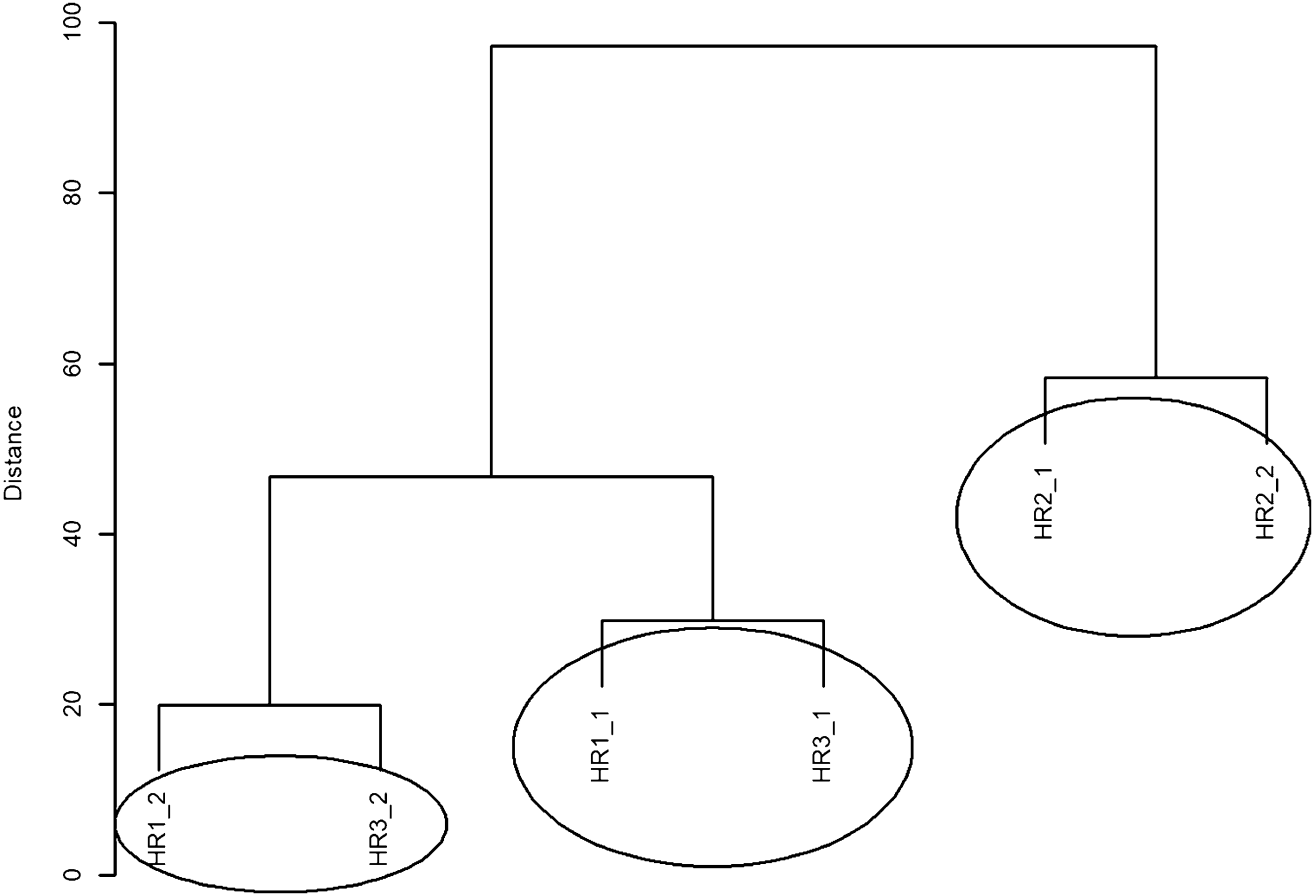
A dendrogram of similarities between the HR values in the CO group based on hierarchical cluster analysis according to the complete clustering method.

The dendrogram of hierarchical clustering with Euclidean measure and complete method for HR in the CO group is shown in Fig. 4. Three different groups containing similar values can be distinguished. In order from the weakest to the strongest effect, these are as follows: the group containing HF1_2 and HF3_2 values, the next group containing HF1_1 and HF3_1 values, and another group consisting of HF2_1 and HF2_2 values.

An analysis of the HF (High Frequency) data was also performed in the CO group. After applying the non-parametric Friedman test for the coupled data, a p-value < 0.0001 was obtained. This means that there is a statistically significant difference between the compared HF objects. Then, for further detailed conclusions, the Nemenyi multiple test was performed and the obtained p-values are shown in Table 7. Statistically significant differences occur between HF1_1 and HF3_1, HF1_1 and HF2_2, HF1_1 and HF3_2, HF1_2 and HF2_2, HF1_2 and HF3_2.

**Table 7:** P-values of the Nemenyi test of HF for the CO group.

	HF1_1	HF2_1	HF3_1	HF1_2	HF2_2
HF2_1	NS	–	–	–	–
HF3_1	**0.017**	NS	–	–	–
HF1_2	NS	NS	NS	–	–
HF2_2	**< 0.001**	NS	NS	**0.003**	–
HF3_2	**0.001**	NS	NS	**0.014**	NS

Values in bold—statistically significant differences, NS—no statistically significant differences.

**Figure 5:**
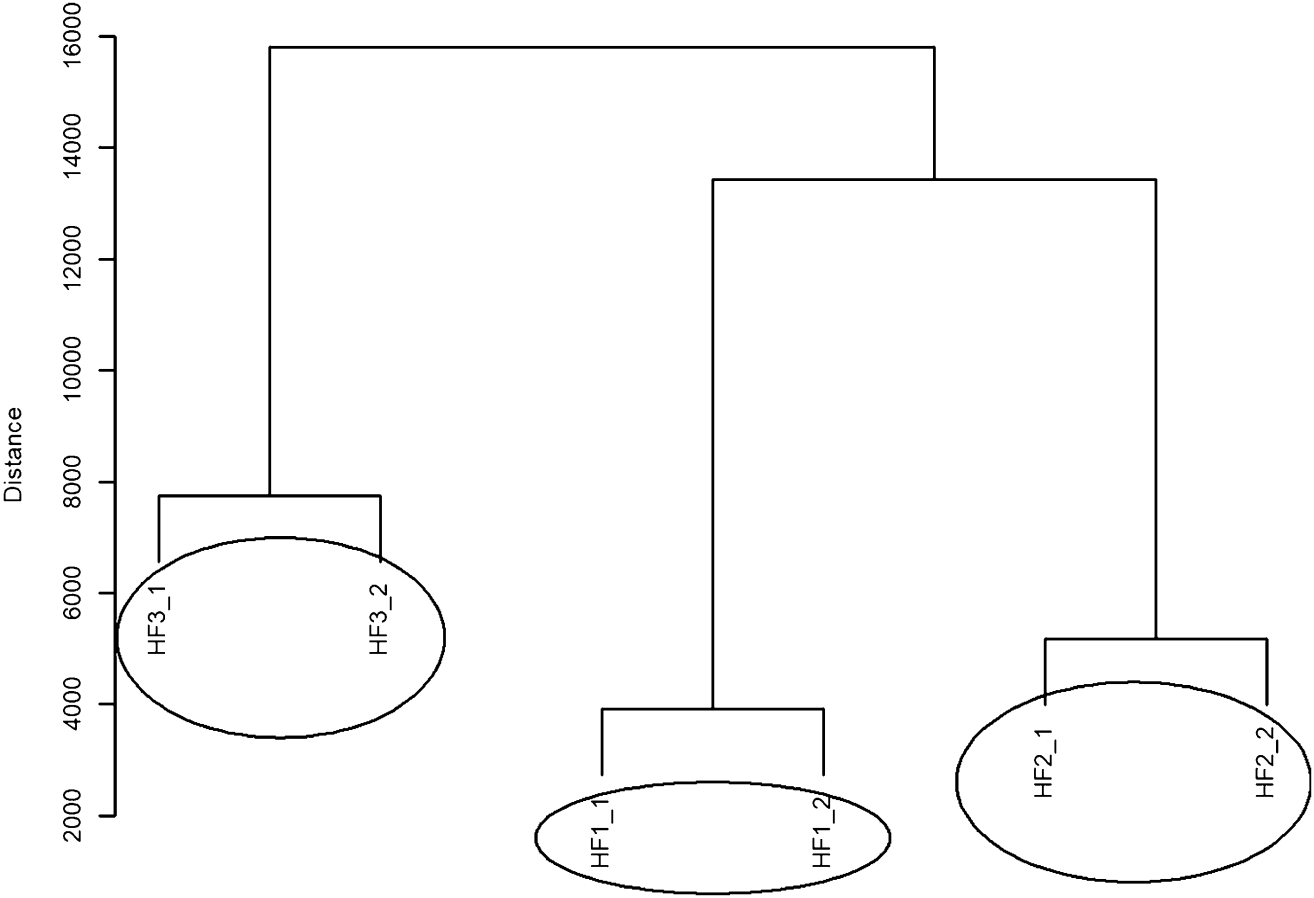
A dendrogram of similarities between the HF values in the CO group based on hierarchical cluster analysis according to the complete clustering method.

Figure 5 shows the dendrogram of hierarchical grouping with Euclidean measure and complete method for HF values in the CO group. Three different groups containing similar values can be distinguished in this dendrogram. In order from the weakest to the strongest effect, these groups are as follows: a group containing HF1_1 and HF1_2 values, the next group containing HF2_1 and HF2_2 values and another group consisting of HF3_1 and HF3_2 values.

The last category of objects analysed in the CO group were LF (Low Frequency) values of heart rate variability. After applying the non-parametric Friedman test for coupled data, a p-value < 0.0001 was obtained. This means that there is a statistically significant difference between the compared LF values (objects). Then, for further de-tailed conclusions, the Nemenyi multiple test was performed and the obtained p-values are shown in Table 8. Statistically significant differences occur between LF1_1 and LF2_1, LF1_1 and LF2_2, LF2_1 and LF_2, LF3_1 and LF2_3, LF1_2 and LF2_2 and LF2_2 and LF3_2 values.

**Table 8:** P-values of the Nemenyi test of LF for the CO group.

	LF1_1	LF2_1	LF3_1	LF1_2	LF2_2
LF2_1	**< 0.001**	–	–	–	–
LF3_1	NS	NS	–	–	–
LF1_2	NS	**0.004**	NS	–	–
LF2_2	**< 0.001**	NS	**0.002**	**< 0.001**	–
LF3_2	NS	NS	NS	NS	**0.021**

Values in bold—statistically significant differences, NS—no statistically significant differences.

**Figure 6:**
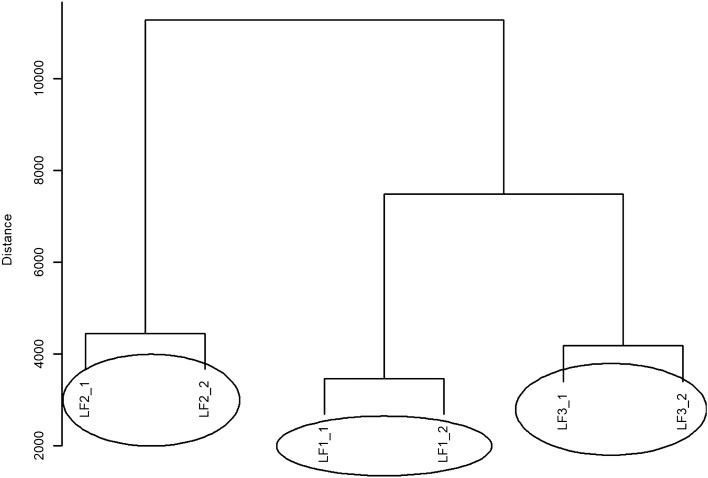
A dendrogram of similarities between the LF values in the CO group based on hierarchical cluster analysis according to the complete clustering method.

The dendrogram showing the hierarchical grouping with Euclidean measure and complete method for LF values in the CO group is presented in Fig. 6. The same three different groups containing values (objects) similar to those in Fig. 5 can be distinguished in this dendrogram. In order from the weakest to the strongest effect, these groups are as follows: the group containing HF1_2 and HF1_2 values, the next group containing HF2_1 and HF2_2 values and another group consisting of HF3_1 and HF3_2 values.

## Discussion

HRV variability can be used to assess stress and mental health objectively^29^. HRV measurements can reflect cardiac activity and the general health of the autonomic system^29^. Many physical conditions can influence HRV values, e.g. physiological factors such as breathing, circadian rhythms, and posture, as well as non-modifiable factors like gender and age, and lifestyle modifiable factors such as physical activity, smoking and medication use, e.g. anticholinergics and beta drugs^29^. Thus, based on current knowledge, it can be said that heart rate (HR) is an indicator of the normal regulation of the human body that effectively adapts to environmental and psychological changes^30–34^. Reduced heart rate variability (HRV) is associated not only with poor cardiovascular health outcomes^35–37^ and vascular disease^38–42^, but also with various mental disorders and cognitive impairments^43–50^.

The results obtained point to rejecting the null hypothesis, which stated that craniosacral techniques do not increase and/or decrease HRV values in male firefighter cadets, and thus point to accepting the alternative hypothesis.

Our study showed that in the study group that received the therapy, it affected the values of HR (Heart Rate) and LF (Low Frequency) of heart rate variability, a statistically significant difference in the p-value < 0.0001 was obtained for these values (Tables 4, 5; Figs. 1, 2, 3). According to Force, the HRV power spectrum can be divided into four bands: ultra-low frequency (ULF; ≤ 0.003 Hz), very low frequency (VLF; 0.0033–0.04 Hz), low frequency (LF; 0.04–0.15 Hz) and high frequency (HF; 0.15–0.4 Hz)^51^. The inherent differences in their signalling mechanisms, the parasympathetic (vagal) modulation of HR, including respiratory sinus arrhythmia (RSA), is faster than the sympathetic activities. Thus, research suggests that HF and LF are prominent reflections of parasympathetic and sympathetic activity, respectively^52^. The short-term periodic changes of HR are also influenced by the cyclic activation of the baroreceptors—a mechanical sensor that works to maintain a homeostatic level of blood pressure^53^. Rapid changes in HR reflect the cardiac regulatory influences of the autonomic nervous system (ANS) in tandem with its dynamic interaction with cardiovascular and respiratory activities^35,51,54,55^.

In our study, manual craniosacral techniques were used, and although manual therapies are offered chiefly for chronic tension, some research studies have recommended the use of this therapeutic modality for increasing patients’ general well-being and suppressing anxiety^56–59^. Firefighters are members of a professional group particularly exposed to high anxiety and a form of stress called traumatic stress, resulting from participation in events in which their physical safety is often threatened^60–62^. High anxiety and stress are associated with accelerated heart rate^63^. Girsberger et al. proved the influence of craniosacral techniques on the reduction of autonomic nervous system tension^64^.

Subjects in the CS group reported relaxation and a better sense of well-being after undergoing cranial techniques, possibly related to improved blood supply to the brain. Shi et al. conducted a study using cranial techniques and obtained an improvement in blood saturation in the prefrontal lobes among subjects^65^. ANS tension can also be reduced by another very well-known and widely practised form of hand treatment, which is therapeutic head massage^66^.

Also in the control group (without cranial techniques), there were statistically significant differences for the values of HR, LF and HF (p-value < 0.0001) (Tables 6, 7, 8; Figs. 4, 5, 6). In our study, in the control group, only a gentle grip on the head was used, which also resulted in a decrease in HRV values, just as in the group in which craniosacral techniques were used. However, the use of deep touch in the temporomandibular joint area increased HRV values^67^. The results from our own study and that of Darren et al. should be explained by modulation of interoception, which is defined as a body-brain sensation axis relating to the visceral body condition^68^.

In addition to being a modulator of interoceptive accuracy, the vagus nerve appears to be strongly associated with how the human organism functions^69^. Interoceptive accuracy (IAc) and vagal tone have a very complex relationship with touch, pain perception and also affective mental health states^67,69^. Stimulation of the left vagus nerve (a component of the parasympathetic system) causes a reduction in heart rate. The vagus nerve is also involved in heart–brain communication. Henley et al. who used muscle-fascia osteopathic techniques in the cervical spine in their study, obtained a reduction in autonomic system tension and thus a reduction in heart rate variability, explaining the result by modulation of the vagus nerve^70^. Cranial techniques have the effect of reducing heart rate reactivity and skin conductance reactivity and thus lowering stress levels^71,72^. An imbalance of the autonomic system, especially a decrease in the activity of part of the parasympathetic system is related to the occurrence of anxiety, post-traumatic stress disorder, depression and schizophrenia^73,74^. The human body uses the phenomenon of allostasis to maintain internal homeostasis and ensure proper functioning of the autonomic system under the influence of changing physiological and environmental stresses^75,76^. The polyvagal theory allows us to understand the reciprocity of effect and bidirectional communication between the heart and the nervous system, through the control of visceral structures i.e. heart, bronchi, thymus and stomatognathic structures (facial and head muscles) by cranial nerves V, VII, IX, X, XI^77^. The polyvagal theory links the evolution of the autonomic nervous system for affective experiences, emotional expression, facial expressions, vocal communication and social behaviour. The theory provides explanations for behavioural, social, emotional and communication disorders^77,78^. Primary regulation in mammals of the vagus heart has shifted from a nonmyelinated pathway originating from the dorsal motor nucleus of the vagus nerve, including myelinated pathways originating from the nucleus ambiguus. The myelinated vagus pathway acts as an active vagus nerve inhibitor, in which rapid inhibition and deinhibition of vagus nerve tension in the heart can rapidly mobilise or calm a person. The heart is strongly influenced by the vagus nerve, via the sinus node^77,78^.

Performing craniosacral techniques, just like applying gentle touch, can be an inexpensive and non-invasive way to reduce HRV and stress itself among people who are occupationally exposed to high levels of stress. However, the application of craniosacral techniques requires further research on a larger group of people.

It is certain that a limitation of this study was its lack of reference to gender-based comparisons. Another limitation was the small study group, which indicates that the study should be expanded to include different occupational groups burdened by stress in their work and to pay attention to workers in a wider age range than presented.

## Conclusions

The study confirmed that craniosacral therapy has an impact on lowering HRV values. Thus, the stated aims of the study were also confirmed, namely that craniosacral therapy and touch can be a low-cost, non-invasive way of lowering HRV values.

Summing up, we have demonstrated that cranial techniques might exert a beneficial effect on heart rate variability in respect of HR and LF values (causing a decrease in heart rate variability values). Statistically significant differences exist but not between all tested values for HR and LF.

Furthermore, in the control group under touch we observed changes in heart rate variability in HR and LF (causing a decrease in heart rate variability values). Moreover, in the control group there are statistically significant differences but not between all values for HR, HF and LF.

It should be acknowledged that there was a lack of an equivalent female group to estimate the effect of sex in this study. In addition, the small size of the groups could be another limitation; therefore, these issues should be investigated with more group participants included in future studies.

As statistically significant values were obtained in both groups, it would be advisable to continue the study to determine which factor has a greater effect on HRV values, whether it be craniosacral therapy or just touching.

## Data Availability

Data and materials are available from the correspondent author.
